# Draft genome sequence data of the osmotolerant yeast *Starmerella magnoliae* JH110 isolated from a honeycomb

**DOI:** 10.1016/j.dib.2026.112565

**Published:** 2026-02-07

**Authors:** Eun-Hee Park, Jeong-Ah Yoon, Min-Kyung Ju, Jin-Ho Seo, Myoung-Dong Kim

**Affiliations:** a Department of Food Science and Biotechnology, Kangwon National University, Chuncheon 24341, Republic of Korea; b Institute of Fermentation and Brewing, Kangwon National University, Chuncheon 24341, Republic of Korea; c Department of Food Biotechnology, Kangwon National University, Chuncheon 24341, Republic of Korea; d Department of Agricultural Biotechnology, Seoul National University, Seoul 08826, Republic of Korea

**Keywords:** *Starmerella magnoliae*, Draft genome, Osmotolerant yeast, Honeycomb

## Abstract

Erythritol is a sugar alcohol used as a sweetener in the food industry and is primarily produced through the fermentation of osmotolerant yeasts and fungi. In this study, we analyzed the genome of *Starmerella magnoliae* JH110 (MDK-2023a), an osmotolerant yeast isolated from a honeycomb and known for producing erythritol. The genome of JH110 was sequenced using the PacBio RS II platform and assembled into four contigs with a total length of 10,358,530 bp and a GC content of 58.5%. Gene prediction was carried out using the MAKER pipeline, and genes for glyoxalase 1 (*GLO1*), transketolase 1 (*TKL1*), and erythrose reductase (*ER*) were identified on the same contig. Phylogenetic analyses based on ITS, BUSCO-derived single-copy core genes, as well as *GLO1* and *TKL1* genes, confirmed that JH110 clustered with other *S. magnoliae* strains, forming a distinct branch separate from other yeasts. This dataset provides genomic information on *S. magnoliae* JH110. It is a valuable resource for comparing the genomes of osmotolerant yeasts, studying sugar alcohol biosynthesis, and exploring potential industrial applications.

Specifications Table

**Table:** 

Subject	Biology
Specific subject area	Genomics and molecular biology
Type of data	Table and Figure
Data collection	Genomic DNA of *Starmerella magnoliae* JH110 (MDK-2023a) was extracted using the i-genomic BYF DNA Extraction Kit (iNtRON, Korea) and then sequenced using the PacBio RS II. De novo assembly was performed using FALCON (v0.5). Gene prediction and annotation were conducted with MAKER (v2.31.8) and UniProt Swiss-Prot (20,100,506).
Data source location	Isolation source: Honeycomb City/Town/Region: Gyeonggi-do Country: Republic of Korea
Data accessibility	Repository name: National Center for Biotechnology Information (NCBI) Data identification number BioProject: PRJNA305085 BioSample: SAMN35302203 SRA: SRR10812052 GenBank: JAUNZF000000000.1 GenBank assembly: GCA_030762955.1 Direct URL to data BioProject: https://www.ncbi.nlm.nih.gov/bioproject/PRJNA305085/ BioSample: https://www.ncbi.nlm.nih.gov/biosample/35302203 SRA: https://www.ncbi.nlm.nih.gov/sra/SRR10812052 GenBank: https://www.ncbi.nlm.nih.gov/nuccore/JAUNZF000000000.1 GenBank assembly: https://www.ncbi.nlm.nih.gov/datasets/genome/GCA_030762955.1/
Related research article	S.Y. Kim, S.S. Park, J.H. Seo, Y.J. Jeon, Analysis of fermentation characteristics for production of erythritol by *Candida* sp., Korean J. Food Sci. Technol. 28 (1996) 935–939.

## 1 Value of the Data

• This study provides the draft genome sequence of *Starmerella magnoliae* JH110 (MDK-2023a), an osmotolerant yeast isolated from a honeycomb, providing essential genomic information for further biological and biotechnological research, such as comparative analysis of erythritol-associated genes and elucidation of their evolutionary relationships among osmotolerant yeasts.
• The sequencing data are publicly accessible in NCBI GenBank and can be used for comparative genomics, identification of functional genes, and validation of traits associated with osmotolerance.
• This dataset is a valuable resource for microbiologists and biotechnologists investigating osmotolerant yeasts, supporting uses in fermentation, sugar alcohol production, and industrial strain development.

## 2 Background

Erythritol is a naturally occurring sugar alcohol that has become popular as a sugar substitute [1]. It is found in various fruits, vegetables, and fermented foods, and is used in food production for its mild, cooling sweetness and moisture-retaining properties [2]. Industrial production of erythritol is primarily carried out through microbial fermentation, where osmotolerant yeasts such as *Yarrowia lipolytica, Moniliella pollinis*, and *Starmerella magnoliae* convert various carbon sources into erythritol [2,3].

*S. magnoliae*, formerly classified as *Candida magnoliae*, was reclassified into the genus *Starmerella* after phylogenetic analyses of the large subunit (LSU) rRNA D1/D2 domain demonstrated that certain *Candida* species form a monophyletic lineage with *Starmerella* [4].

*S. magnoliae* was isolated from a honeycomb and designated JH110 at Seoul National University. The same strain was obtained at Kangwon National University, where it was assigned the internal stock designation MDK-2023a. *S. magnoliae* JH110 (MDK-2023a) is an osmotolerant yeast that produces erythritol from sugars such as fructose, glucose, and sucrose [5,6]. It produces erythritol and glycerol as compatible osmolytes to cope with hyperosmotic stress caused by up to 50 % (w/v) sugar concentration [7]. Previous studies have identified the transketolase 1 (*TKL1*), glyoxalase 1 (*GLO1*), and erythrose reductase (*ER*) genes in *S. magnoliae*, which are involved in erythritol biosynthesis and osmotic-stress responses [6,8,9].

In this study, we obtained the genome sequence of *S. magnoliae* JH110 (MDK-2023a) and carried out a comparative genomic analysis. This dataset provides a foundation for understanding the genetic basis of its metabolic traits and for exploring potential applications in fermentation and sugar-related industries.

## 3 Data Description

**Fig. 1:**
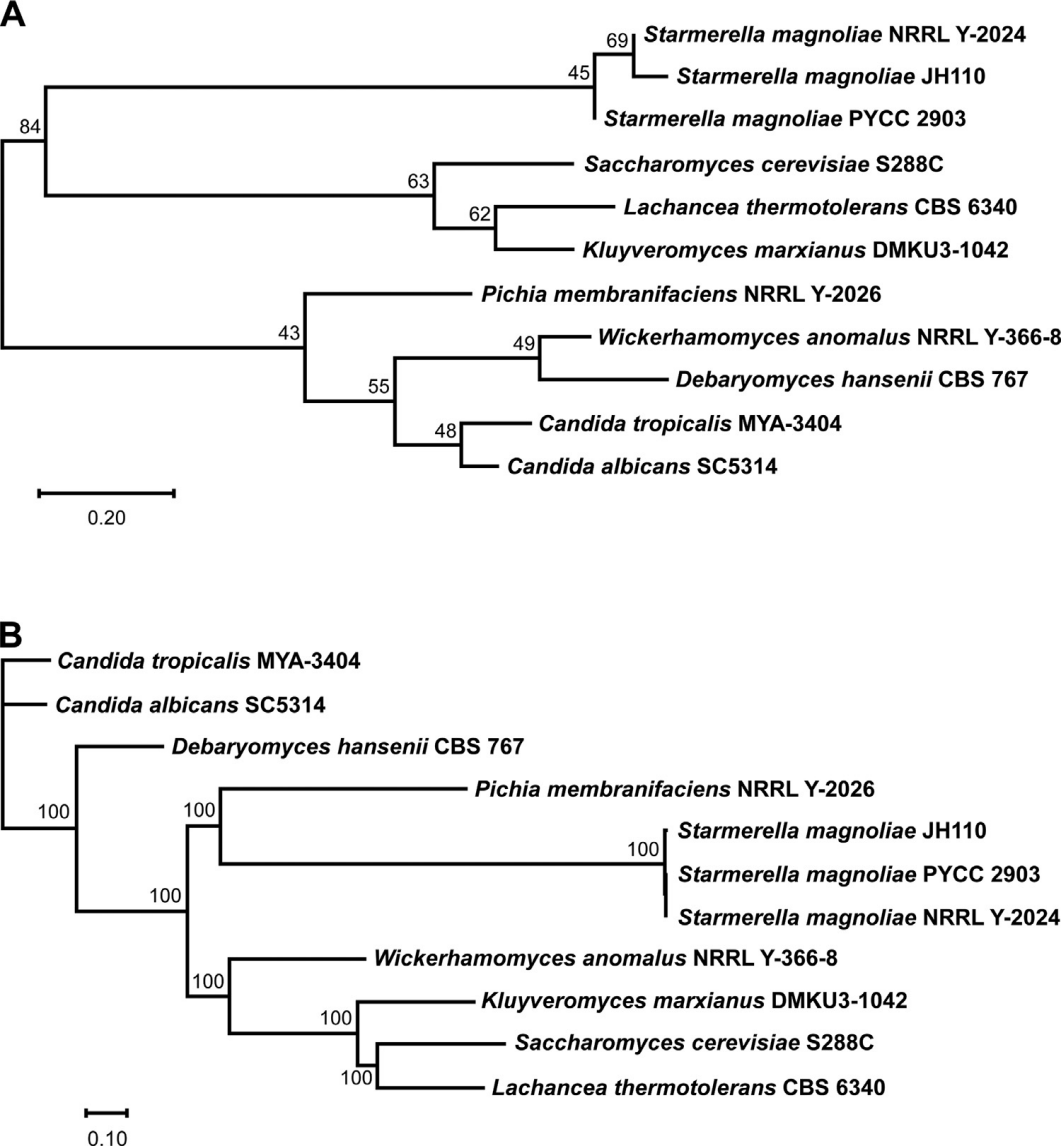
Phylogenetic tree of *Starmerella magnoliae* JH110 and other yeast strains based on (A) the ITS region and (B) the core genes. Numbers at the nodes indicate bootstrap values, and the scale bar represents relative branch lengths.

In the phylogenetic trees based on the ITS region (Fig. 1A) and BUSCO-derived single-copy core genes (Fig. 1B), S*. magnoliae* JH110 (MDK-2023a) clustered with *S. magnoliae* PYCC 2903 and NRRL Y-2024. In both trees, JH110 (MDK-2023a) and the other *S. magnoliae* strains formed a distinct branch, separate from other yeast strains.

Table 1 summarizes the genomic features of *S. magnoliae* JH110 (MDK-2023a) in comparison with other yeast strains. The genome of *S. magnoliae* JH110 (MDK-2023a) was assembled into four contigs with a total size of 10,358,530 bp, a GC content of 58.5 %, and 7,903 predicted genes. The genome sizes of the other yeast species ranged from 10,920,159 bp to 14,579,835 bp, with GC contents between 33.0 % and 47.5 %.

**Table 1:** Genomic features of *Starmerella magnoliae* JH110 and other yeast strains.

Strain	Genome size (bp)	Number of contigs	N50	GC content (%)	GenBank accession number
*Starmerella magnoliae* JH110	10,358,530	4	3,299,905	58.5	GCA_030762955.1
*Starmerella magnoliae* PYCC 2903	9,801,260	1,021	27,508	58.5	GCA_003033435.1
*Starmerella magnoliae* NRRL Y-2024	10,224,282	633	58,172	58.5	GCA_030579715.1
*Candida tropicalis* MYA-3404	14,579,835	23	1,654,078	33.0	GCF_000006335.3
*Candida albicans* SC5314	14,282,666	8	2,231,883	33.5	GCF_000182965.3
*Debaryomyces hansenii* CBS 767	12,152,486	7	2,007,515	36.5	GCF_000006445.2
*Pichia membranifaciens* NRRL Y-2026	11,582,150	10	1,676,949	45.0	GCF_001661235.1
*Wickerhamomyces anomalus* NRRL Y-366–8	14,145,566	46	2,188,197	34.5	GCF_001661255.1
*Kluyveromyces marxianus* DMKU3–1042	10,920,159	8	1,421,472	40.0	GCF_001417885.1
*Saccharomyces cerevisiae* S288C	12,071,326	16	924,431	38.5	GCF_000146045.2
*Lachancea thermotolerans* CBS 6340	10,392,862	8	1,513,537	47.5	GCF_000142805.1

**Fig. 2:**
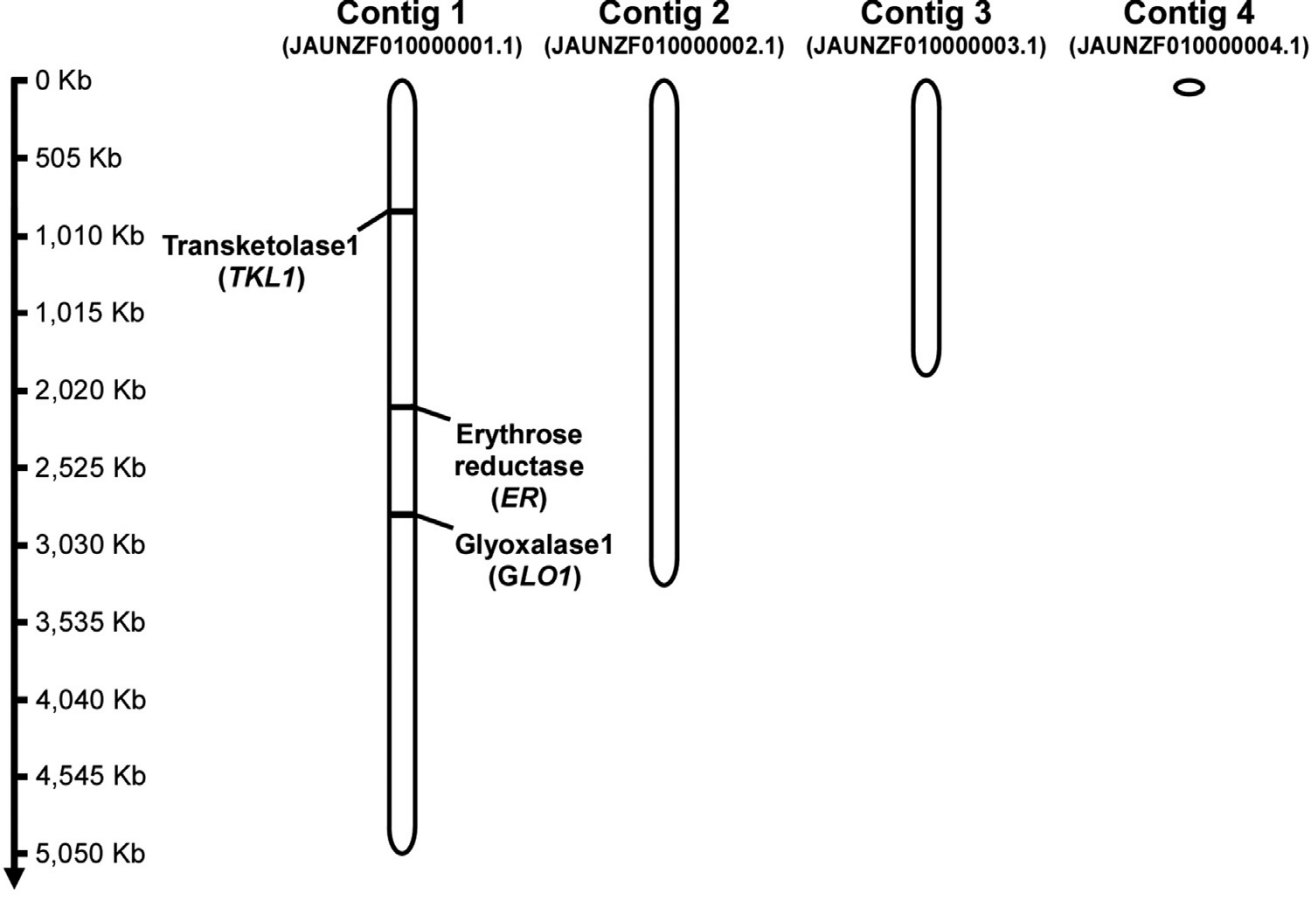
Genomic locations of the *GLO1, TKL1*, and *ER* genes in the *Starmerella magnoliae* JH110 genome.

**Fig. 3:**
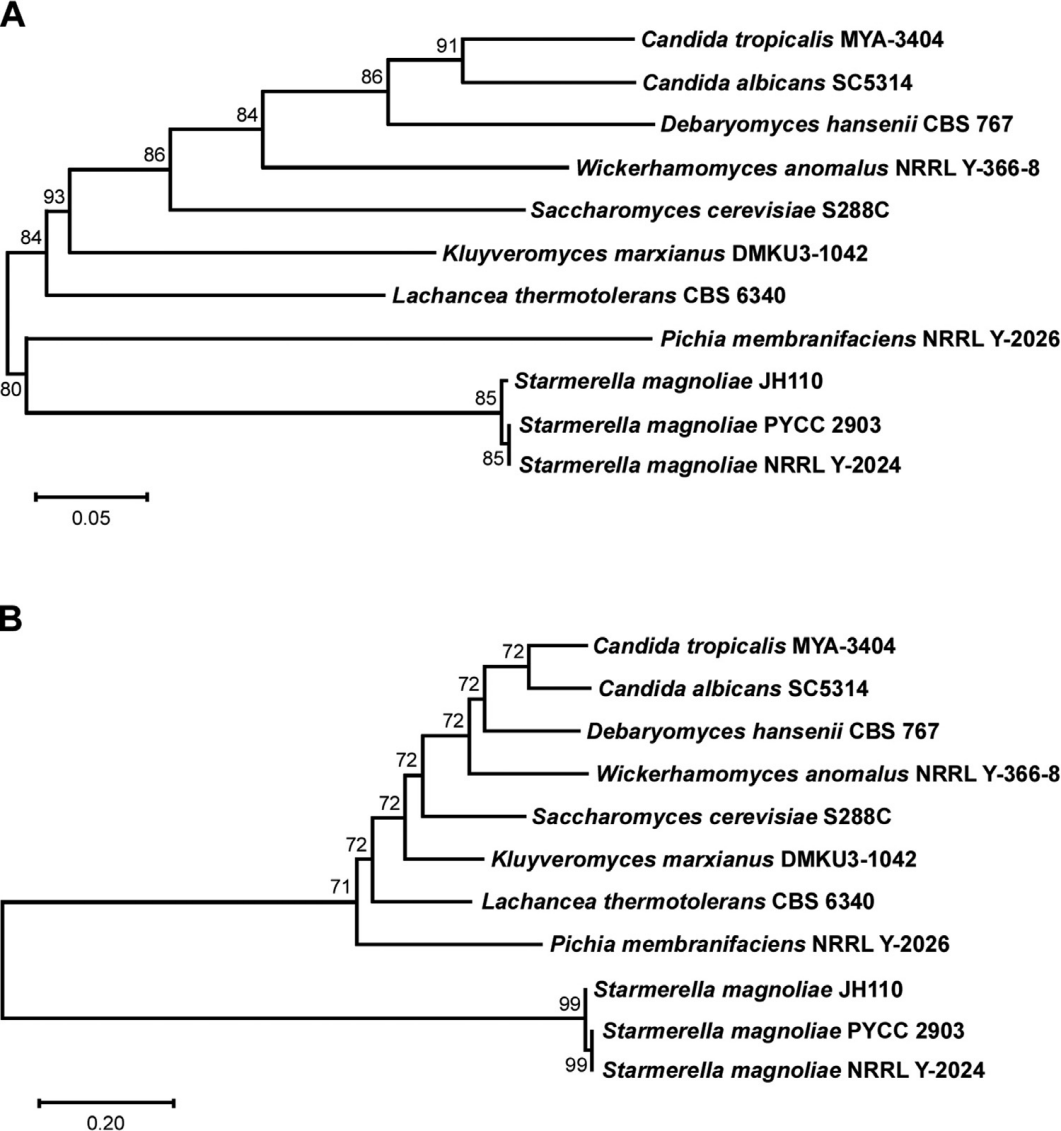
Phylogenetic tree of *Starmerella magnoliae* JH110 and other yeast strains based on (A) the *GLO1* and (B) the *TKL1* genes. Numbers at the nodes show bootstrap values, and the scale bar indicates relative branch lengths.

In the assembled genome of *S. magnoliae* JH110 (MDK-2023a), four linear contigs were obtained with lengths of 5,050,565 bp, 3,299,905 bp, 1,929,375 bp, and 78,685 bp. The *GLO1, TKL1,* and *ER* genes were all located on contig 1 (Fig. 2). Phylogenetic analysis based on the *GLO1* (Fig. 3A) and *TKL1* (Fig. 3B) genes showed that JH110 (MDK-2023a) clustered with *S. magnoliae* PYCC 2903 and NRRL Y-2024, forming a distinct branch separate from other yeast strains.

## 4 Experimental Design, Materials, and Methods

Approximately 1 g of honeycomb, collected in Gyeonggi-do, Korea, was inoculated into 100 mL of liquid medium containing 10 g/L yeast extract and 400 g/L glucose, followed by incubation at 30°C with shaking at 200 rpm for 24 h. The culture was spread onto solid medium composed of 10 g/L yeast extract, 200 g/L glucose, and 20 g/L agar, and incubated at 30°C for 48 h to obtain a single colony. The single colony was cultivated in YEPD liquid medium at 30°C for 24 h. Genomic DNA was extracted using the i-genomic BYF DNA Extraction Kit (iNtRON, Korea) according to the manufacturer’s instructions. The isolate was identified by amplifying and sequencing the internal transcribed spacer (ITS) region using the primers ITS1 (5′-TCCGTAGGTGAACCTGCGG-3′) and ITS4 (5′-TCCTCCGCTTATTGATATGC-3′), followed by sequence similarity analysis using the BLAST tool of the National Center for Biotechnology Information (NCBI)**.**

The genome of *S. magnoliae* JH110 (MDK-2023a) was sequenced using PacBio RS II (Pacific Biosciences, USA). The reads were *de novo* assembled into four contigs using FALCON (v0.5) [10]. Gene prediction was conducted using MAKER (v2.31.8) [11], and protein BLAST was performed against the UniProt Swiss-Prot database (20,100,506) [12]. The genome of *S. magnoliae* JH110 (MDK-2023a) was visualized using MapGene2Chromosome (MG2C v2.1) [13].

For comparative genomic analysis, osmotolerant yeast strains deposited in the National Center for Biotechnology Information (NCBI) as reference genomes and annotated for the *GLO1* and *TKL1* genes were selected. Phylogenetic trees were generated from BUSCO-derived single-copy core genes and the internal transcribed spacer (ITS) region, *GLO1*, and *TKL1* genes. BUSCO (v5.5.0) [14] was used to identify orthologs, which were then analyzed with IQ-TREE (v2.2.2) [15] using the maximum-likelihood method. Sequences of ITS, *GLO1*, and *TKL1* were aligned and analyzed using MEGA12 [16] with the neighbor-joining method.

## Limitations

Not applicable.

## Ethics Statement

The authors have read and followed the ethical requirements for publication in Data in Brief and have confirmed that the current work does not involve human subjects, animal experiments, or any data collected from social media platforms.

## CRediT authorship contribution statement

**Eun-Hee Park:** Conceptualization, Investigation, Data curation, Writing – original draft, Project administration. **Jeong-Ah Yoon:** Conceptualization, Formal analysis, Investigation, Data curation, Writing – original draft, Writing – review & editing, Visualization, Project administration. **Min-Kyung Ju:** Data curation, Visualization. **Jin-Ho Seo:** Writing – review & editing. **Myoung-Dong Kim:** Conceptualization, Writing – review & editing, Supervision, Project administration.

## Data Availability

NCBIdraft genome of Starmerella magnoliae (Original data).

## References

[1] Mazi T.A., Stanhope K.L. (2023). Erythritol: an in-depth discussion of its potential to be a beneficial dietary component. Nutrients.

[2] Khatape A.B., Dastager S.G., Rangaswamy V. (2022). An overview of erythritol production by yeast strains. FEMS Microbiol. Lett..

[3] Cheng H., Wang S., Bilal M., Ge X., Zhang C., Fickers P., Cheng H. (2018). Identification, characterization of two NADPH-dependent erythrose reductases in the yeast *yarrowia lipolytica* and improvement of erythritol productivity using metabolic engineering. Microb. Cell Fact..

[4] Santos A.R.O., Leon M.P., Barros K.O., Freitas L.F.D., Hughes A.F.S., Morais P.B., Lachance M.A., Rosa C.A. (2018). *Starmerella* camargoi f.a., sp. nov., *Starmerella* ilheusensis f.a., sp. nov., *Starmerella* litoralis f.a., sp. nov., *Starmerella* opuntiae f.a., sp. nov., *Starmerella* roubikii f.a., sp. nov. And *Starmerella* vitae f.a., sp. nov., isolated from flowers and bees, and transfer of related *Candida* species to the genus *Starmerella* as new combinations. Int. J. Syst. Evol. Microbiol..

[5] Kim S.Y., Park S.S., Seo J.H., Jeon Y.J. (1996). Analysis of fermentation characteristics for production of erythritol by *Candida* sp. Korean J. Food Sci. Technol..

[6] Lee D.H., Lee Y.J., Ryu Y.W., Seo J.H. (2010). Molecular cloning and biochemical characterization of a novel erythrose reductase from *Candida* magnoliae JH110. Microb. Cell Fact..

[7] Yu J.H., Lee D.H., Oh Y.J., Han K.C., Ryu Y.W., Seo J.H. (2006). Selective utilization of fructose to glucose by *Candida* magnoliae, an erythritol producer. Appl. Biochem. Biotechnol..

[8] Yoo B.H., Park E.H., Seo J.H., Kim M.D. (2014). Cloning of the transketolase gene from erythritol-producing yeast *Candida* magnoliae. J. Microbiol. Biotechnol..

[9] Park E.H., Lee D.H., Seo J.H., Kim M.D. (2011). Cloning and characterization of a glyoxalase I gene from the osmotolerant yeast *Candida* magnoliae. J. Microbiol. Biotechnol..

[10] Chin C.S., Peluso P., Sedlazeck F.J., Nattestad M., Concepcion G.T., Clum A., Dunn C., O'Malley R., Figueroa-Balderas R., Morales-Cruz A., Cramer G.R., Delledonne M., Luo C., Ecker J.R., Cantu D., Rank D.R., Schatz M.C. (2016). Phased diploid genome assembly with single-molecule real-time sequencing. Nat. Methods.

[11] Holt C., Yandell M. (2011). MAKER2: An annotation pipeline and genome-database management tool for second-generation genome projects. BMC Bioinform..

[12] Consortium U. (2010). The universal protein resource (UniProt) in 2010. Nucleic. Acids. Res..

[13] Chao J., Li Z., Sun Y., Aluko O.O., Wu X., Wang Q., Liu G. (2021). MG2C: A user-friendly online tool for drawing genetic maps. Mol. Hortic..

[14] Manni M., Berkeley M.R., Seppey M., Simão F.A., Zdobnov E.M. (2021). BUSCO update: novel and streamlined workflows along with broader and deeper phylogenetic coverage for scoring of eukaryotic, prokaryotic, and viral genomes. Mol. Biol. Evol..

[15] Minh B.Q., Schmidt H.A., Chernomor O., Schrempf D., Woodhams M.D., von Haeseler A., Lanfear R. (2020). IQ-TREE 2: new models and efficient methods for phylogenetic inference in the genomic era. Mol. Biol. Evol..

[16] Kumar S., Stecher G., Suleski M., Sanderford M., Sharma S., Tamura K. (2024). MEGA12: Molecular evolutionary genetic analysis version 12 for adaptive and green computing. Mol. Biol. Evol..

